# Report on the Physiology and Pathology of the Blood

**Published:** 1846-10

**Authors:** 


### ECLECTIC DEPARTMENT,

### AND SPIRIT OF THE MEDICAL PERIODICAL PRESS.

---

*Report on the Physiology and Pathology of the Blood.*

The study of the blood in health and disease, at the present moment, is of great interest and importance to the pathologist and practitioner. Medicine is emerging from the restraints of a too exclusive solidism, and (as we have reason to hope,) is advancing with a rational, humoral pathology, *pari passu*, in its scientific character, and in its practical resources. 'A better acquaintance with the composition of the blood, and the morbid changes to which it is liable, has already produced an immense improvement in the pathological aspects and relations of several diseases. Compare our present knowledge of the philosophy of inflammation, anæmia, plethora, diabetes, morbus Brightii, for examples, with the views entertained respecting these affections when the attention of pathologists was limited to the lesions of the solid structures of the organism! Compare, too, the influence of improved pathological doctrines, in the same instances, upon medical practice!

In view of the intimate connection of the study of the blood with that of a host of morbid conditions, we know of no department of physiology and pathology which has greater claims upon the attention of the physician. On this account, the following report, presenting a compendium of the more important principles appertaining to the blood in its healthy state, and in its diverse perversions, will, we think, be acceptable to our readers. We are indebted for the report to our esteemed contemporary the Illinois and Indiana Med. and Surg. Journal. Its author, as we recognise by the initials appended, is Prof. Herrick, one of the editors of that Journal. In a subsequent number of the Journal the subject is continued by a consideration of the condition of the blood in Bright's disease of the Kidneys. This we do not deem it necessary to transfer to our columns, as the very valuable series of papers by our correspondent

Dr. Geo. N. Burwell, contains a full enunciation of the important changes which the blood undergoes in that disease.—*Editor Buffalo Medical Journal.*

"The following remarks upon the elaboration, composition, and relative constituent proportions of the blood; without professing to comprise a synopsis, even, of the most recently developed facts and opinions upon this subject, are presented to our readers for the purpose, merely, of calling their attention to the subject, and of showing the importance of making the very remarkable pathological changes that take place in this fluid, a means of diagnosticating and treating diseases.

It is a well known fact, that nutritious substances taken as food, are, by the process of digestion converted into a fluid substance called chyle, the composition of which is albumen and saline matters dissolved in water, with fat globules suspended in the fluid.

In its passage from the intestines, through the lacteals, to the mesenteric glands, and thence to the thoracic duct, the chyle undergoes many and important changes; the most remarkable of which is the conversion of its albumen into another organic principle called fibrine. "Albumen, then, may be regarded as the first proximate element; at the expense of which in conjunction with fatty matter all the tissues of the animal body are ultimately formed. Still this organic principle, as long as it remains in a state regarded by chemists as characteristic of it; exhibits no tendency to become organized, and it is only when it has been subjected to certain peculiar vital influences, and, perhaps, undergone a change in its chemical constitution, or, in other words, has been converted into fibrine, that any such tendency manifests itself.

As the consequence of this change, the fibrine increases, and the albumen lessens, whilst, at the same time, the oil-globules undergo an evident diminution. It is not conceivable that the fibrine should be at once formed from the oil-globules, since albumen is much more ready to undergo organization and vitalization, and is always the preceding principle to fibrine. The fibrine must, therefore, be produced at the expense of the albumen, whilst new albumen is elaborated from the oily matter.

The following table presents a view of the relative proportions of the three principal ingredients in the chyle in different parts of the absorbent system, and gives an idea of its advance in the progress of assimilation.

| | |
|---|---|
| In lacteals, from intestines, to mesenteric glands. | Fat in maximum quantity.<br>Albumen in minimum quantity.<br>Fibrine almost entirely wanting. |
| In lacteals, from mesenteric glands to thoracic duct. | Fat in medium quantity.<br>Albumen in maximum quantity.<br>Fibrine in medium quantity. |

| | |
|---|---|
| In thoracic duct. | Fat in minimum quantity.<br>Albumen in medium quantity.<br>Fibrine in maximum quantity. |

The fluid, drawn from the thoracic duct is frequently observed to present a decided red tinge, which increases on exposure to air. This tinge is due to the presence of true blood corpuscles, smaller, however, than those of the true sanguineous fluid.

As to the composition of blood then, it may be said to be composed of water holding albumen, fibrine and saline matters in solution, having particles of a red color floating in the fluid. These, the true blood globules, are flattened discs, having a circular outline and composed of three parts, the capsule, the nucleus, and the contained matter, thus constituting a complete nucleated cell. It is a peculiarity in the composition of these globules that they each contain a small proportion of iron: the red color of the blood is due to its presence.

Blood drawn from the body, and left to itself, soon presents a new arrangement of its organic elements. The fibrine coagulates and separates from the fluid portion, attracting and enclosing in its meshes the red globules, thus forming what is called the coagulum or clot. The remaining portion, designated serum, is composed of water holding in solution the albumen and saline matters.

According to M. Andral, the usual or normal proportions of these different ingredients in the blood, are as follows:

| | | |
|---|---|---|
| Of each element in 1000 parts, | Fibrine, | 3 |
| | Globules, | 127 |
| | Solid matter, dissolved in serum, | 80 |
| | Water, | 790 |

The proportion of fibrine may vary in health from 2.5 to 3.5, that of the globules from 110 to 140. The clot formed by the coagulation of blood, being dependent for firmness upon the fibrine, and for color and volume upon the amount of globules, indicates, by its density and more perfect reticulated structure, an exuberance of fibrine, and by its softness, bulk and high color, a comparatively large proportion of globules.

Blood coagulated slowly, and in which the proportion of fibrine is large, as compared with the globules, permits the coloring matter to settle to the bottom of the clot, thus leaving a nearly colorous layer of fibrine upon its surface, long since known to physicians and described by writers as the *buffy coat*. In cases where the proportion of fibrine is large, and the coagulum firm, it presents a concave or cupped surface.

All the organs of the body being dependent upon the blood for nutrition and support, are, consequently, deranged in their functions and subject to change of structure whenever this fluid becomes deteriorated in quality, or changed as to the relative normal quantities of its constituent proportions.

Among the most important and easily detected pathological changes which take place in the blood, are those resulting from a want of the due proportion of its elements, such as an exuberance or deficiency of globules, increased or diminished amount of fibrine, wa t of albumen, saline matters, &c.

Of the abnormal conditions and diseases of the system, in which such changes in the constituent proportions of the blood are most apparent, may be enumerated Plethora, Anæmia, Pyrexiæ, Phlegmasiæ, Hemorrhages and Dropsies.

Contrary to the generally received opinion that the peculiar condition of the system in plethoric individuals depends upon exuberance of blood, it appears from the researches of Andral, that the marked peculiarity of this fluid in the above named condition of the system, is an increase in the amount of its globules. In 31 bleedings of plethoric subjects, in each of which an analysis of the blood was made by this distinguished pathologist, the average amount of globules in 1000 parts was 141, the normal quantity being 127. Hence it is that the blood of such persons is high colored, presenting, after coagulation, a comparatively small amount of serum, a voluminous clot filled with fluid, and soft for want of fibrine, but never incrusted with the buffy coat.

Coincident with an excess of globules in the blood of the plethoric, there are certain physiological and pathological phenomena dependent, as it seems, upon this peculiar condition of the fluid. All the functions of life are more active, digestion quick, respiration full, circulation rapid, with a degree of general excitability of the whole organism; thus showing that the blood of plethoric individuals is highly exciting, and of a stimulating character. This truth is made more apparent, also, by the fact, that such persons are subject to vertigo, dizziness, ringing in the ears, &c., symptoms produced, without doubt, by the passage of an excessive number of globules through the brain. Plethoric persons are, also, more liable than others to active hemorrhages and appoplectic effusions, the result, evidently, of the absence of the due proportion of coagulable matter in the blood.

By blood-letting, abstemiousness, and general antiphlogistic treatment, the number of blood-globules are readily diminished.

It appears, then, that an excess of the globular element of the blood is a constant distinguishing characteristic of plethora, a fact leading to the inference of that, which is in reality true, viz: that in anæmia,—a condition of the system directly opposite to that of plethora,—the amount of globules fall far below the natural standard.

This anæmic condition of the system, is characterised by paleness of countenance, general muscular debility, nervousness, neuralgia, vertigo, dyspnœa, and palpitations of the heart upon the slightest effort.

An example of spontaneous anæmia, in which most of the above named symptoms are always present, is that not uncommon but peculiar disease of females. chlorosis.

This want of the globular element in the blood is often the effect of debilitating diseases and profuse hemorrhages. Pregnant women, also, during the last months of gestation, often become anæmic. Andral observes, "I have found as the average of the proportion of globules in 16 cases of commencing anæmia, the cipher 109, and in 24 cases of confirmed anæmia, the cipher 65"— 127 as above stated being the normal quantity.

The physical properties of the blood drawn from anæmic patients is perfectly characteristic of its condition. For want of globules the coagulum is small, and swims in a large amount of perfectly colorless serum; but, as there is no want of fibrine, it is firm and generally presents on its surface a well-marked buffy coat, resulting from the absence of globules in this upper stratum of the fibrinous mass.

Thus it is seen that the presence of the buffy coat is not always a diagnostic sign of inflammation, since it appears upon the blood of patients whose general condition, evidently, requires a course of treatment directly the opposite to that, generally considered to be most efficacious in the phlegmasiæ.

In order to increase the amount of blood globules and thus restore the blood to its normal condition, those means are to be employed which are generally believed to give tone and vigor to the system, such as nutritious food, fresh air, exercise and tonics, of which, the preparations of iron are most effectual.

As an example of the good effects of proper treatment in such diseases, Andral mentions the case of a young lady, who, in 3 months, had the amount of globules so much increased as to present a change in that short space of time, from a confirmed anæmic to a well-marked plethoric condition.

The *pyrexiæ* are another class of diseases in which the change in the blood, if not so well marked and defined, are still of great practical importance as indications for diagnosis and treatment.

These affections being characterized like the phlegmasiæ, by general febrile excitement, an attempt has been made, by several distinguished writers, to make it appear that the fever attendant upon them is always symptomatic of local disease, and that they should all be classed together with simple inflammatory affections.

"Such pretensions," remarks Andral, "cannot be maintained. The pyrexiæ exist as diseases apart, the causes which often develop them, the symptoms which characterize them, the special nature of the alterations which they produce in the solids, are already enough of grave reasons for not confounding the pyrexiæ and phlegmasiæ;" "but the analysis of the blood comes still more to establish a very remarkable difference between the one and the other class," for "in the phlegmasiæ there are always two constant alterations which march together, that of the solid and that of the blood, it is no longer the same in the pyrexiæ; in these diseases in reality, the only phenomenon which never fails, is the fever itself."

But although it is true as above stated, that the pyrexiæ may exist without any appreciable alteration in the solid or fluids, yet there never is in these, as in inflammatory diseases, an increase in the amount of fibrine. On the contrary, this organic element, even in the milder forms of these diseases, is generally diminished, and in well marked cases, it is not only uniformly lessened in quantity, but becomes deteriorated in quality, as appears from the imperfect structure of its coagulum.

Blood drawn from an individual suffering under the influence of typhus, typhoid, miasmatic fevers, or other forms of pyrexiæ, coagulates slowly. The fibrine, being small in quantity, and of an inferior quality, the clot is voluminous, imperfectly separated from the serum, soft and easily broken; in fine, the blood of such persons presents appearances indicative of low vitality, and a want of coagulable matter.

In the above described morbid condition of this fluid, there being a diminished amount of fibrine, but, at the same time, a normal quantity, or a relatively increased proportion of globules, the buffy coat, resulting, as before stated, either from an exuberance of the former, or a paucity of the latter element, is never present.

This affords another important distinguishing characteristic between the phlegmasiæ and pyrexiæ. "I may affirm," says Andral, "that I have never met with it (the buff) unless there was phlegmasial complication, either in slight or severe typhoid fever, in measles, in scarlatina, or in variola."

As the result of a want of fibrine and a normal tendency to coagulation in the blood in the advanced stages of the adynamic or putrid type of the pyrexiæ, it has a tendency to exude from its vessels; hence the effusion of blood into the pustules in variola, the hemorrhages in malignant scarlatina, and the epistaxis and bleeding of the gums in the advanced stages of severe typhus and typhoid fevers.

The numerous sanguineous congestions in this class of diseases, mistaken often, for inflammations, are evidently the effects of the absence of a due proportion of coagulable matter in the blood. The enlargement and softening of many organs, as the spleen, are also, the effect, many times, of an accumulation of such blood in their structure.

As to the origin of the pyrexiæ, it may be said, that it is by no means probable that the want of fibrine is the primary cause of these diseases, for it seems incontestible, "that the specific cause which gives them birth, acts upon the blood in such a way, that it tends to destroy its spontaneously coagulable matter." "If this cause acts with slight energy, or if the economy resists it, the destruction of the fibrine is not accomplished; if, on the contrary, the cause continue to act with all its intensity, and the forces of the organism be in fault, the destruction of the fibrine will commence either at the very beginning of the disease, which is very rare, or at a certain period after its commencement: all this applies itself equally well both to the typhoid fevers, and the eruptive fevers."*

It is a fact well known to the profession, that there are but few diseases of the class pyrexiæ, for which we have remedies of known efficacy; and it is equally true, that of the means of restoring the blood, in these diseases, to its normal condition, we are equally uninformed. It has been suggested by some, that the administration of such agents as favour the coagulation of blood, such as acids, &c., might prove beneficial.—[*To be continued.*]

